# Pseudotime-Based derivation of a PET-Based metabolic progression index for prognostic stratification in Extensive-Stage SCLC

**DOI:** 10.1007/s12149-026-02162-8

**Published:** 2026-02-04

**Authors:** Ayşegül Aksu, Anılcan Us, Zeynep Gülsüm Güç, Kadir Alper Küçüker, Ahmet Alacacıoğlu, Bülent Turgut

**Affiliations:** 1 Department of Nuclear Medicine, İzmir Kâtip Çelebi University Atatürk Training and Research Hospital, İzmir, Turkey; 2 Department of Oncology , İzmir Kâtip Çelebi University Atatürk Training and Research Hospital , İzmir, Turkey

**Keywords:** Metabolic progression index, SCLC, DmaxVox, PET/CT, Pseudotime

## Abstract

**Purpose:**

To develop and validate a pseudotime-derived imaging biomarker integrating volumetric, metabolic, and spatial information from baseline 18 F-Fluorodeoxyglucose Positron Emission Tomography/Computed Tomography (18 F-FDG PET/CT) for prognostic stratification in patients with extensive-stage small cell lung cancer (SCLC).

**Methods:**

This retrospective study included 83 patients with stage IV SCLC who underwent baseline 18 F-FDG PET/CT prior to systemic therapy. Whole-body tumor lesions were semi-automatically segmented, and conventional PET-derived metrics reflecting tumor burden, metabolic activity, inter-lesional heterogeneity, and spatial dissemination were extracted. A diffusion map–based manifold learning framework was applied to derive a latent progression axis, termed the Metabolic Progression Index (MPI), representing a pseudotime-based continuum of metastatic disease severity. The primary endpoint was 12-month all-cause mortality. Prognostic performance of MPI was evaluated using logistic regression, receiver operating characteristic (ROC) analysis, and internal bootstrap validation, and compared with conventional PET-derived parameters.

**Results:**

During 12-month follow-up, 60 patients (72.3%) died. MPI showed strong correlations with spatial dissemination and moderate correlations with tumor burden metrics, without being fully explained by any single conventional parameter. In multivariable analysis, MPI emerged as an independent predictor of 12-month mortality (OR per 0.1-unit increase = 1.87, 95% CI: 1.41–2.62; *p* < 0.001). The MPI-based model demonstrated superior discriminative performance compared with conventional PET-derived models, with significant incremental prognostic value confirmed by likelihood ratio testing.

**Conclusion:**

A pseudotime-based integration of baseline 18 F-FDG PET/CT features captures a latent continuum of metastatic disease architecture in extensive-stage SCLC. MPI provides prognostic information beyond conventional PET-derived metrics and may serve as a robust imaging biomarker for risk stratification.

## Introduction

Small cell lung cancer (SCLC) is an aggressive neuroendocrine malignancy accounting for approximately 15% of all lung cancers and is characterized by rapid proliferation, early systemic dissemination, and poor long-term outcomes [1]. Despite high initial response rates to platinum-based chemotherapy, most patients relapse early, and prognosis remains poor, particularly in extensive-stage disease [2]. In stage IV SCLC, median overall survival rarely exceeds one year, even in the era of chemo-immunotherapy, highlighting the need for robust prognostic biomarkers capable of capturing the complexity of advanced metastatic disease [3].

Whole-body 18 F-Fluorodeoxyglucose Positron Emission Tomography/Computed Tomography (18 F-FDG PET/CT) plays a central role in staging and baseline assessment of SCLC by enabling simultaneous evaluation of tumor metabolism and disease extent. Quantitative PET-derived parameters such as metabolic tumor volume (MTV) and total lesion glycolysis (TLG) have demonstrated prognostic value across multiple malignancies, including lung cancer [4, 5]. More recently, spatial dissemination metrics—most notably Dmax, defined as the maximum distance between tumor lesions—have emerged as markers of metastatic aggressiveness [6–8]. However, these parameters are often highly correlated and provide overlapping information [9]. For instance, MTV and TLG typically exhibit near-perfect collinearity, while Dmax reflects only a single geometric extreme of spatial spread. As a result, their combined use in multivariable models is statistically challenging and may lead to unstable or misleading prognostic inferences.

More fundamentally, conventional radiomic approaches typically treat imaging features as independent predictors, selecting subsets through regression-based frameworks [10, 11]. This strategy may be suboptimal in metastatic SCLC, where tumor burden, metabolic activity, spatial dissemination, and inter-lesional heterogeneity represent interdependent manifestations of a common underlying process—systemic disease progression. Rather than attempting to isolate individual features, an alternative approach is to integrate these dimensions into a single composite descriptor of overall metastatic severity.

Computational frameworks originally developed in single-cell biology, particularly pseudotime analysis and manifold learning, provide a principled methodology for this task [12–15]. When applied to PET-derived volumetric, metabolic, and spatial features, these approaches enable extraction of a dominant axis of radiological variation that reflects a continuous gradient of metastatic severity across patients, while mitigating multicollinearity and reducing dimensionality without reliance on arbitrary feature selection. In this setting, pseudotime denotes a data-driven ordering along a latent imaging axis rather than chronological disease progression.

In this study, we used diffusion map–based manifold learning to derive an outcome-agnostic composite imaging biomarker from baseline whole-body 18 F-FDG PET/CT, termed the Metabolic Progression Index (MPI). We evaluated the prognostic value of MPI for 12-month mortality in patients with stage IV SCLC and compared its performance with conventional PET-derived metrics, individually and in optimized multivariable models.

## Materials and methods

### Study population

Patients with histopathologically confirmed SCLC who underwent diagnostic or staging 18 F-FDG PET/CT were retrospectively reviewed. Patients were eligible for inclusion if they met the following criteria: age 18 years or older; stage IV SCLC detected at PET/CT; no previous treatment for lung cancer; availability of baseline 18 F-FDG PET/CT images and survival data. Cerebral/cerebellar metastases were not considered for lesion segmentation because of the high physiological 18 F-FDG uptake in the brain parenchyma [16, 17].

Clinical and demographic data were collected from the hospital information system. In addition, anatomical distribution and metastatic patterns were recorded, including primary tumor laterality (right or left lung), presence of lymph node, bone, liver, and adrenal metastases, as well as stage IV subclassification (stage IVA vs. IVB) according to the 9th edition of the TNM classification. Overall survival (OS) was calculated from the date of pathological diagnosis to the date of death from any cause. For secondary descriptive and classification-based analyses, patients were additionally dichotomized using a 12-month survival threshold.

Additionally, patients were categorized into four groups according to the systemic treatments they received: (1) standard therapy (platinum + etoposide), (2) standard therapy combined with immunotherapy (platinum + etoposide plus either durvalumab or atezolizumab), (3) alternative regimens (platinum-based therapies without etoposide, non-platinum chemotherapies, or immune checkpoint inhibitor monotherapy), and (4) no systemic treatment (patients without oncology referral or systemic therapy initiation).

### 18 F-FDG PET/CT imaging and image analysis

PET/CT was performed in accordance with the European Association of Nuclear Medicine (EANM) guideline for patients whose blood glucose level was found to be below 150 ng/dl after at least 4 h of fasting [18]. For each patient, intravenous injection of an average of 0.1 mCi/kg (3.7 MBq/kg) 18 F-FDG was administered. An average of 60 ± 10 min after the injection of the activity, 18 F-FDG PET/CT was performed with a combined PET/CT scanner (Discovery 710 PET/CT Scanner, GE Medical Systems, Waukesha, Wisconsin, USA) with three-dimensional mode. The voxel size was 4 × 4 × 4 mm and the emission scans were obtained with a position of 8–10 beds per patient and for 2 min/bed position from vertex to mid-thigh. Transmission scans using CT with 300 mA and 120 kVp were obtained. Ordered-subset expectation maximization algorithm with 3 iterations and 24 subsets was used as the reconstruction method.

**Fig. 1:**
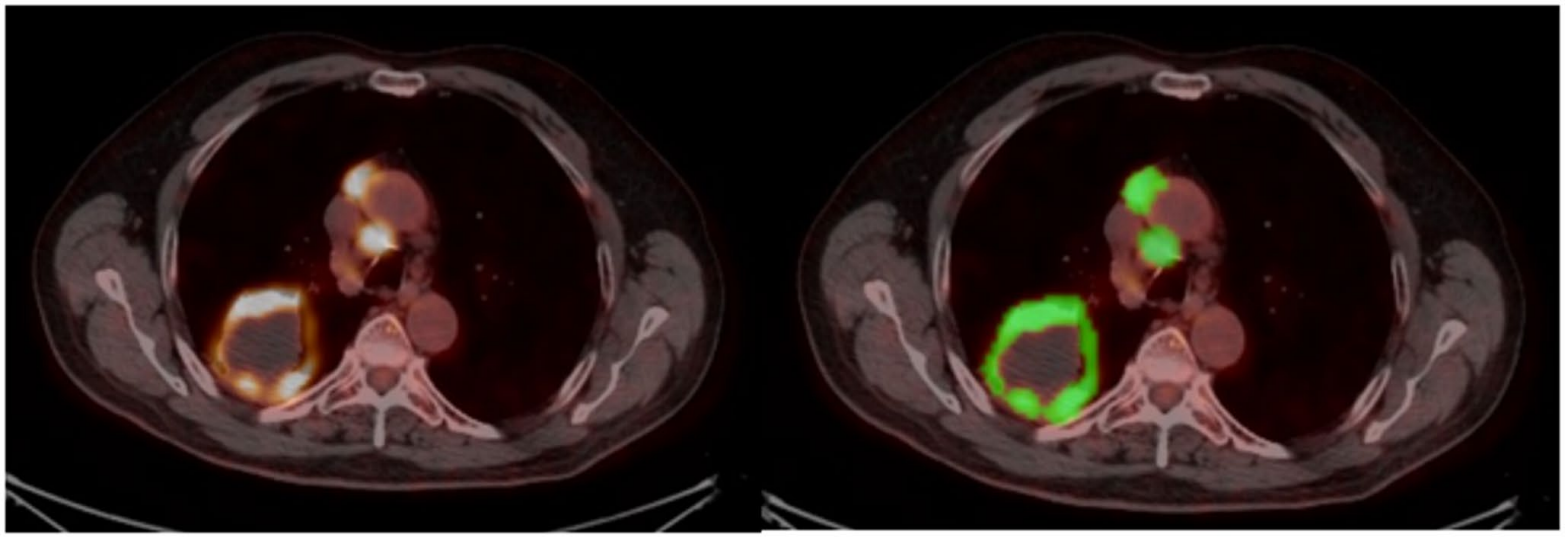
Representative examples of lesion segmentation and volumetric/dissemination metrics derived from pre-treatment 18 F-FDG PET/CT. *Left panel*: Fused PET/CT images of a patient showing a primary lung mass with mediastinal lymph node metastases. *Right panel*: Corresponding semi-automatic segmentation of metabolically active tumor lesions using a fixed SUV threshold of 2.5, with volumes of interest (VOIs) displayed in green

All pre-treatment 18 F-FDG PET/CT images in DICOM format were analyzed using LIFEx software (version 7.6) [19]. Lesions were segmented semi-automatically using a fixed SUV threshold of 2.5, including only foci with uptake above this threshold while excluding physiological uptake areas (Fig. 1) [16, 20, 21]. Although fixed-threshold segmentation may underestimate lesions with low 18 F-FDG uptake, the application of a uniform threshold across all patients ensured internal methodological consistency for relative comparisons and latent trajectory modeling. Segmentation accuracy was visually verified on corresponding CT images to ensure correct anatomical localization.

For each segmented volume of interest (VOI), a comprehensive set of quantitative metabolic, volumetric, and spatial features was extracted. To enhance interpretability and reduce redundancy in feature description, PET-derived parameters were conceptually grouped into four categories reflecting complementary aspects of metastatic disease burden.

First, global tumor burden metrics characterized the overall volumetric and metabolic extent of disease and included total metabolic tumor volume (tMTV), total lesion glycolysis (tTLG), and cumulative volume and intensity-based measures. MTV was defined as the total volume of voxels with an SUV greater than 2.5 within each VOI, while TLG was calculated by multiplying MTV by the corresponding SUVmean [16].

Second, dominant-lesion descriptors captured features of the largest or most metabolically active lesion, including the volume of the largest (bulk) lesion and the smallest lesion, as well as bulk-to-other lesion volume and intensity difference metrics.

Third, inter-lesional heterogeneity indices quantified variability in lesion size and metabolic activity across metastatic deposits, incorporating differences between the highest and lowest lesion volumes and SUVmax values, as well as cumulative inter-lesional difference sums. Finally, spatial dissemination parameters described the anatomical spread of disease; Dmax and DmaxVox, defined as the maximum Euclidean distance between the two most distant voxels across all segmented lesions. DmaxVox was preferentially employed as a dissemination metric, as centroid-based distances may underestimate the true spatial extent of disease in patients with large or irregularly shaped lesions. By incorporating voxel-level extremes, DmaxVox provides a more anatomically faithful estimate of metastatic spread. The features used for MPI calculation are detailed in Table 1. All image-derived features were extracted by a board-certified nuclear medicine physician who was blinded to clinical outcomes.

**Table 1:** Summary of PET-derived features utilized for MPI construction

Feature Category	Parameter Name (LIFEx)	Description	Biological Rationale
**Global Tumor Burden**	tMTV	Total Metabolic Tumor Volume; sum of all segmented lesion volumes.	Reflects the cumulative systemic tumor mass.
	tTLG	Sum of Total Lesion Glycolysis (MTV × SUVmean) across all lesions.	Integrates volumetric burden with cumulative metabolic activity.
	Intensity_Sum	Cumulative sum of voxel intensities (SUV) within all ROIs.	Measures the total magnitude of tracer uptake.
	MeanIntensity_Sum	Sum of mean intensities of all segmented ROIs.	Represents the average metabolic load of the entire disease.
	Volume_intensity_sum	Sum of the products of voxel volumes and intensities.	Provides an integrated assessment of tumor mass and density.
**Dominant Lesion Descriptors**	MTV_Bulk	Volume of the largest segmented ROI.	Represents the primary tumor or the most dominant metastatic site.
	MTV_Smallest	Volume of the smallest segmented ROI.	Defines the lower limit of detectable metastatic foci.
	tSUVmax	Maximum SUVmax observed across all lesions.	Indicates the peak metabolic aggressiveness of the disease.
	MaxIntensity_Min	Minimum SUVmax observed among all segmented lesions.	Defines the lower threshold of metabolic activity in the tumor set.
**Inter-lesional Heterogeneity**	Volume_DiffSum	Sum of volume differences between all pairs of ROIs.	Quantifies size-based architectural variability across metastases.
	Bulk_Volume_DiffSum	Cumulative volume difference between the largest ROI and all other ROIs.	Measures the imbalance between the dominant site and disseminated disease.
	BulkSmallest_Volume_Diff	Difference in volume between the largest and the smallest ROIs.	Reflects the dynamic range of metastatic lesion sizes.
	MaxIntensity_DiffSum	Sum of SUVmax differences between all pairs of ROIs.	Represents metabolic phenotype diversity across different sites.
	HighestLowest_MaxIntensity_Diff	Difference between the highest and lowest SUVmax values.	Captures the metabolic spectrum of the whole-body tumor burden.
	Bulk_MaxIntensity_DiffSum	Sum of SUVmax differences between the largest ROI and all others.	Highlights metabolic disparity between the primary site and metastases.
**Spatial Dissemination**	DmaxVox	Maximum Euclidean distance between the two furthest voxels.	Provides a high-precision estimate of the anatomical spread.
	Dmax	Maximum distance between the centroids of the two furthest ROIs.	Measures the global geometric extent of systemic disease.
	BulkDmax	Maximum distance between the largest ROI and any other ROI.	Contextualizes the spatial relationship of metastases to the primary site.
	BulkCentroidCoor_DistMax	Maximum distance from the centroid of the largest ROI to others.	Represents the reach of systemic spread from the dominant lesion.
	SmallestCentroidCoor_DistMax	Maximum distance from the centroid of the smallest ROI to others.	Reflects the spatial isolation or spread of minor metastatic clusters.

MTV: Metabolic Tumor Volume; TLG: Total Lesion Glycolysis; SUV: Standardized Uptake Value; ROI: Region of Interest; Dmax: Maximum distance between lesion centroids; DmaxVox: Maximum Euclidean distance between the two furthest voxels

## Metabolic progression index Estimation

To model a latent continuum of metastatic disease severity, trajectory inference analysis was performed using a diffusion map–based nonlinear dimensionality reduction framework [22]. The resulting latent variable was defined as the MPI, representing a pseudotime-derived geometric ordering along a latent imaging manifold that integrates metabolic burden, spatial dissemination, and inter-lesional heterogeneity, rather than temporal disease progression.

To avoid circularity with survival outcomes, only baseline, pre-treatment whole-body PET-derived parameters were used for MPI construction. These parameters encompassed the four conceptual feature categories described above: (i) global volumetric and metabolic tumor burden metrics; (ii) dominant-lesion descriptors; (iii) inter-lesional heterogeneity indices; and (iv) spatial dissemination parameters, including both centroid- and voxel-based distance measures (Dmax and DmaxVox). Individual features were not analyzed independently but were jointly integrated to derive a latent representation of phenotypic disease severity across patients.

Prior to manifold learning, all volumetric, metabolic, and spatial PET-derived parameters were standardized using feature-wise z-score normalization (mean-centered and scaled to unit variance) to ensure comparability across features with different numeric scales. The standardized feature matrix was subsequently embedded into a low-dimensional nonlinear manifold using diffusion map analysis, implemented with the destiny package in R (version 4.3.3) [23]. Diffusion maps were computed using a k-nearest neighbor graph (k = 20), local adaptive kernel scaling (sigma = “local”), and Euclidean distance. To assess sensitivity to neighborhood size, diffusion map embedding and MPI derivation were repeated using k values from 10 to 30; patient rankings were highly consistent across this range (Spearman’s ρ = 0.995–1.000 relative to the k = 20 embedding), supporting k = 20 as a stable choice balancing local connectivity and manifold smoothness in this cohort.

Diffusion maps were selected because of their ability to preserve local geometric relationships in high-dimensional data and to model smooth, non-linear transitions between complex tumor phenotypes that may not be adequately captured by linear dimensionality reduction methods such as principal component analysis [24].

The first diffusion component (DC1), representing the dominant continuous axis of variation within the radiomic manifold, was interpreted as the principal latent severity coordinate. Higher-order diffusion components were explored but were not used for MPI derivation because they did not demonstrate consistent monotonic ordering or clinically interpretable associations across patients, supporting the selection of DC1 as the primary latent coordinate.

**Fig. 2:**
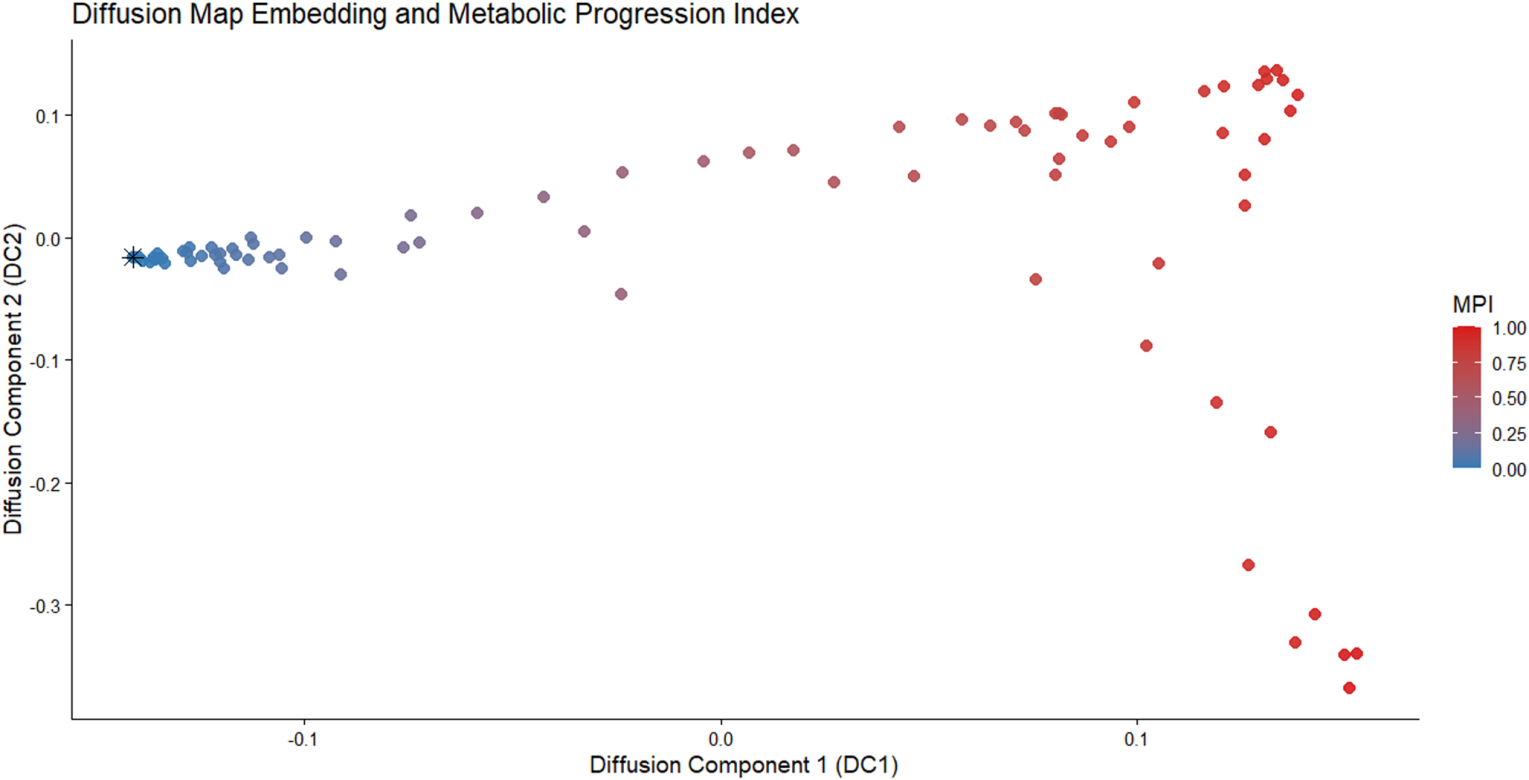
Diffusion map embedding of baseline whole-body 18 F-FDG PET-derived features. Each point represents an individual patient, colored according to the Metabolic Progression Index (MPI). The first diffusion component (DC1) was interpreted as the principal latent metabolic progression axis. The root state corresponds to the patient with minimal global tumor burden and limited spatial dissemination

The trajectory root was defined by selecting the patient with the lowest DC1 value, corresponding to minimal global tumor burden and limited spatial dissemination within the cohort. MPI values were subsequently derived by rank-ordering patients along DC1 and rescaling these values to a continuous 0–1 interval using min–max normalization, thereby preserving the monotonic structure of the inferred latent continuum [22]. This data-driven root selection strategy avoids subjective anchoring and ensures that the inferred ordering reflects the intrinsic geometry of the imaging feature space. The diffusion map embedding and derivation of MPI are illustrated in Fig. 2.

To assess robustness of the inferred ordering, diffusion map embedding and MPI derivation were repeated using 80% subsamples of the cohort across 300 iterations. Stability of patient ordering was quantified using Spearman rank correlation between subsampled and full-cohort MPI rankings.

Sensitivity analyses were also performed to evaluate dependence of MPI on specific feature families. In particular, MPI was re-derived after exclusion of dissemination parameters (Dmax and DmaxVox) from the feature set, and correlations between original and ablated MPI were assessed.

### Statistical analysis

All statistical tests were two-sided, and a p value < 0.05 was considered statistically significant. Statistical analyses were performed using R software (version 4.3.3; R Foundation for Statistical Computing, Vienna, Austria). Continuous variables were summarized as median with range or mean ± standard deviation (SD), as appropriate, based on data distribution. Categorical variables were reported as frequencies and percentages.

The primary clinical endpoint was 12-month all-cause mortality. Patients were dichotomized according to survival status at 12 months following pathological diagnosis into those who died within 12 months and those who survived beyond 12 months. Associations between MPI and conventional PET-derived parameters were evaluated using Spearman’s rank correlation coefficients, given the non-normal distribution and potential non-linear relationships of imaging-derived variables.

Multivariable models were constructed using predefined covariates with strong univariable associations (*p* < 0.05) and low inter-correlation (Spearman’s ρ < 0.7) to mitigate multicollinearity, as assessed by variance inflation factors (VIF). Additional clinical factors (e.g., age, sex, treatment category) were not included in the final models due to lack of univariable significance and minimal confounding effect on the primary imaging parameters.

To assess independent prognostic value, two predefined multivariable logistic regression models were constructed. The conventional multivariable model included the two PET-derived parameters with the strongest univariable associations (tMTV and DmaxVox), together with bone metastasis status as a clinically relevant covariate. The MPI-based multivariable model included MPI and bone metastasis status. Given the strong correlation between MPI and DmaxVox (ρ = 0.84), these variables were not included in the same model to avoid multicollinearity and overadjustment.

Model coefficients were reported as odds ratios (ORs) with corresponding 95% confidence intervals (CIs). MPI values were normalized to a 0–1 range, and ORs were additionally expressed per 0.1-unit increase to facilitate clinical interpretability.

Model discrimination was evaluated using receiver operating characteristic (ROC) curve analysis and quantified by the area under the curve (AUC). Comparisons between ROC curves were performed using DeLong’s test. The incremental prognostic value of MPI beyond conventional PET-derived parameters was formally assessed using likelihood ratio testing by comparing the MPI-based model with a model including bone metastasis status alone. Internal validation was performed using bootstrap resampling with 1,000 iterations to estimate optimism-corrected model performance. Model calibration was assessed using grouped calibration analysis based on predicted risk deciles.

Overall survival was analyzed using the Kaplan–Meier method, and median survival time with corresponding 95% confidence intervals was reported. In addition, overall survival was analyzed using Cox proportional hazards regression with MPI as a continuous covariate. Hazard ratios (HRs) were reported per 0.1-unit increase in MPI. Multivariable Cox models including selected clinical covariates were explored to assess shared prognostic variance. For clinical interpretability, HRs and ORs for MPI were additionally reported per 0.1-unit increase, while regression modeling was performed using the original 0–1 scaled variable.

## Results

### Patient characteristics and outcomes

A total of 83 patients (mean age: 65 ± 10 years; range: 43–84 years) were included in the study. Of the total cohort, 12 (14.5%) were female and 71 (85.5%) were male. During the 12-month follow-up period, 60 patients (72.3%) died, while 23 patients (27.7%) remained alive at or beyond 12 months. According to Kaplan–Meier survival analysis, the median overall survival was 6.2 months (95% CI: 4.1–8.3 months).

### Clinical and Treatment-Related factors and 12-month mortality

Among the clinical and metastatic features, the presence of bone metastasis was significantly associated with 12-month mortality (*p* = 0.019). In contrast, treatment category was not significantly associated with survival status (*p* = 0.074), although a numerical trend toward improved outcomes was observed in patients receiving standard therapy with or without immunotherapy. Prior radiotherapy history showed no association with 12-month survival (*p* = 0.878). Similarly, no significant associations were observed between 12-month mortality and sex, lymph node involvement, liver metastases, adrenal metastases, or M-stage subclassification (*p* > 0.05)(Table 2).

**Table 2:** Baseline clinical and metastatic characteristics of the study cohort stratified by 12-Month survival status

Variable	All Cohort (*n* = 83)	Survival < 12 Months (*n* = 60)	Survival ≥ 12 Months (*n* = 23)	*p* value
**Age (mean ± std**,** range)**	65 ± 10 (43–84)	66 ± 9 (45–84)	63 ± 10 (43–80)	0.297
**Gender** **Female** **Male**	12 61	9 51	3 20	1.000
**Laterality** **Right** **Left**	45 (54.2%) 38 (45.8%)	35 25	10 13	0.224
**Lymph Node Metastasis** **Yes** **No**	81 (97.6%) 2 (2.4%)	59 1	22 1	0.480
**Liver Metastasis** **Yes** **No**	34 (41%) 49 (59%)	28 32	6 17	0.088
**Bone Metastasis** **Yes** **No**	46 (55.4%) 37 (44.6%)	38 22	8 15	0.019
**Adrenal Metastasis** **Yes** **No**	30 (36.1%) 53 (63.9%)	19 41	11 12	0.170
**M Stage** **1a** **1b** **1c1** **1c2**	8 (9.6%) 12 (14.5%) 11 (13.3%) 52 (62.7%)	5 7 9 39	3 5 2 13	0.524
**Stage IVA/IVB** **IVA** **IVB**	20 (24.1%) 63 (75.9%)	12 48	8 15	0.159
**Chemo/immunotherapy** **Standard therapy** **Standard therapy + immunotherapy** **Alternative regimens** **No systemic treatment**	40 5 9 29	26 2 7 25	14 3 2 4	0.086
**Radiotherapy** **Yes** **No**	23 (26.7%) 63 (73.3%)	17 43	6 17	0.838

## Correlation between MPI and conventional PET-Derived parameters

Spearman correlation analysis demonstrated strong associations among conventional PET-derived tumor burden metrics, particularly between tMTV and tTLG (ρ = 0.98, *p* < 0.001), confirming substantial collinearity between these parameters.

MPI showed moderate to strong correlations with volumetric and intensity-based metrics, including tMTV (ρ = 0.70, *p* < 0.001) and tTLG (ρ = 0.66, *p* < 0.001). The strongest association was observed between MPI and DmaxVox (ρ = 0.84, *p* < 0.001), indicating that MPI is closely linked to spatial dissemination patterns in addition to overall tumor burden. Although DmaxVox exhibited the highest individual correlation with MPI, no single conventional imaging parameter fully explained the variability captured by the MPI axis.

Feature-wise correlation analysis with DC1 demonstrated strong associations not only with spatial dissemination metrics (DmaxVox: ρ = 0.89; Dmax: ρ = 0.87) but also with inter-lesional heterogeneity indices (Volume_DiffSum: ρ = 0.88; Bulk_Volume_DiffSum: ρ = 0.87) and global tumor burden parameters (tMTV: ρ = 0.72; tTLG: ρ = 0.70). This distribution of correlations supports the interpretation of DC1—and thus MPI—as an integrative multidimensional descriptor of metastatic disease severity instead of merely rescaling spatial dissemination. Given the high degree of correlation among several imaging-derived parameters, separate multivariable models were constructed to mitigate the risk of multicollinearity.

## Univariable and multivariable associations with 12-month mortality

In univariable analyses, several PET-derived parameters were significantly associated with 12-month mortality (Table 3). MPI demonstrated the strongest association with mortality, followed by tMTV, tTLG and DmaxVox. Due to the high degree of collinearity among volumetric and dissemination metrics, these variables were not entered simultaneously into multivariable models.

**Table 3:** Prognostic performance of PET-Derived imaging parameters for 12-Month mortality

PET-Derived Parameters	All Cohort (*n* = 83)	Survival < 12 Months (*n* = 60)	Survival ≥ 12 Months (*n* = 23)	*p* value	AUC (95% CI)
**MPI**	0.708 (0–1)	0.802 (0.375–1.000.375.000)	0.097 (0–0.934.934)	< 0.001	0.800 (0.671–0.989)
**tMTV**	511.65 (36.60–5387.90.60.90)	576.50 (36.60–5387.90.60.90)	217.37 (43.94–2635.99.94.99)	0.003	0.715 (0.589–0.844)
**tTLG**	2904.77 (234.16–28043.60.16.60)	3243.30 (234.16–28043.60.16.60)	1362.30 (270.95–13039.00)	0.009	0.687 (0.556–0.818)
**Intensity_Sum**	103,625 (9427.35–1.03 × 10^6^)	115841.78 (9577.53–1.03 × 10^6^)	43536.71 (9427.35–525963.12.35.12)	0.007	0.692 (0.559–0.825)
**MeanIntensity_Sum**	41.01 (10.06–147.92.06.92)	50.57 (11.05–147.92.05.92)	30.78 (10.06–99.06)	0.038	0.648 (0.527–0.768)
**Volume x Intensity_Sum**	56,727,545 (350541-3.97.97 × 10^9^)	73,616,865 (350541-3.97.97 × 10^9^)	8,206,338 (414244-1.39.39 × 10^9^)	0.004	0.704 (0.574–0.835)
**MTV_Bulk**	236.18 (35.57–4916.65.57.65)	275.52 (35.57–4916.65.57.65)	143.77 (38.77–1621.17.77.17)	0.013	0.644 (0.546–0.808)
**MTV_Smallest**	0.48 (0.024–70.16)	0.52 (0.02–70.16)	0.32 (0.03–52.24)	0.548	0.543 (0.404–0.681)
**tSUVmax**	15.51 (6.09–73.07)	15.57 (6.09–73.07)	15.42 (9.48–35.50)	0.935	0.506 (0.376–0.635)
**MaxIntensity_Min**	4.50 (2.93–19.89)	4.76 (2.93–17.03)	4.34 (3.01–19.89)	0.404	0.559 (0.419–0.768)
**Volume_DiffSum**	2973.92 (38.48–52085.09.48.09)	4482.24 (47.55–52085.09.55.09)	1019.52 (38.48–21212.56.48.56)	0.006	0.696 (0.564–0.829)
**BulkVolume-DiffSum**	2067.19 (38.48–51473.41.48.41)	3018.98 (47.55–51473.41.55.41)	667.19 (38.48–21048.42.48.42)	0.010	0.683 (0.549–0.818)
**BulkSmallestVolume-DiffSum**	235.83 (35.45–4914.26.45.26)	274.98 (35.45–4914.26.45.26)	143.51 (38.48–1620.85.48.85)	0.011	0.681 (0.550–0.813)
**MaxIntensity_DiffSum**	46.32 (6.18–1294.30.18.30)	50.45 (6.18–1294.30.18.30)	29.03 (12.11–175.78.11.78)	0.211	0.589 (0.465–0.713)
**HighestLowest_MaxIntensity_Diff**	10.28 (2.95–69.21)	10.14 (2.95–69.21)	11.49 (5.34–17.53)	0.515	0.454 (0.327–0.581)
**Bulk_MaxIntensity_DiffSum**	37.44 (5.37–325.97.37.97)	49.82 (5.37–325.97.37.97)	29.03 (12.11–175.78.11.78)	0.242	0.583 (0.456–0.711)
**DmaxVox**	46.56 (7.36–105.0)	53.30 (7.35–105.00)	28.12 (12.54–93.12)	0.031	0.654 (0.526–0.781)
**Dmax**	42.60 (3.65–102.10)	50.01 (3.65–102.10)	25.18 (9.57–90.13)	0.039	0.647 (0.519–0.775)
**Bulk_Dmax**	28.98 (3.65–69.37)	37.29 (3.65–65.24)	20.20 (9.57–69.37)	0.173	0.597 (0.464–0.730)
**BulkCentroidCoor_DistMax**	28.98 (3.65–69.37)	37.29 (3.65–65.24)	20.20 (9.57–69.37)	0.173	0.597 (0.464–0.730)
**SmallestCentroidCoor_DistMax**	97.40 (0.02–70.16)	45.69 (3.65–102.10)	20.56 (9.57–86.26)	0.027	0.658 (0.531–0.785)

MPI: Metabolic Progression Index; tMTV: Total Metabolic Tumor Volume; TLG: Total Lesion Glycolysis; SUV: Standardized Uptake Value; Dmax: Maximum distance between lesion centroids; DmaxVox: Maximum Euclidean distance between the two furthest voxels; AUC: Area Under the Curve; CI: Confidence Interval.

In the primary MPI-based multivariable logistic regression model, which included MPI and bone metastasis status, MPI emerged as a strong independent predictor of 12-month mortality (OR per 0.1-unit increase = 1.87, 95% CI: 1.41–2.62, *p* < 0.001) (Table 4). Bone metastasis status did not retain independent statistical significance after adjustment for MPI (*p* = 0.063), with attenuation of its prognostic contribution in the multivariable model. No evidence of problematic multicollinearity was observed, with VIF values below 3 for all covariates. For clinical interpretability, MPI was scaled to a 0–1 range, and odds ratios were reported per 0.1-unit increase.

### Comparison with conventional PET-Based models

**Fig. 3:**
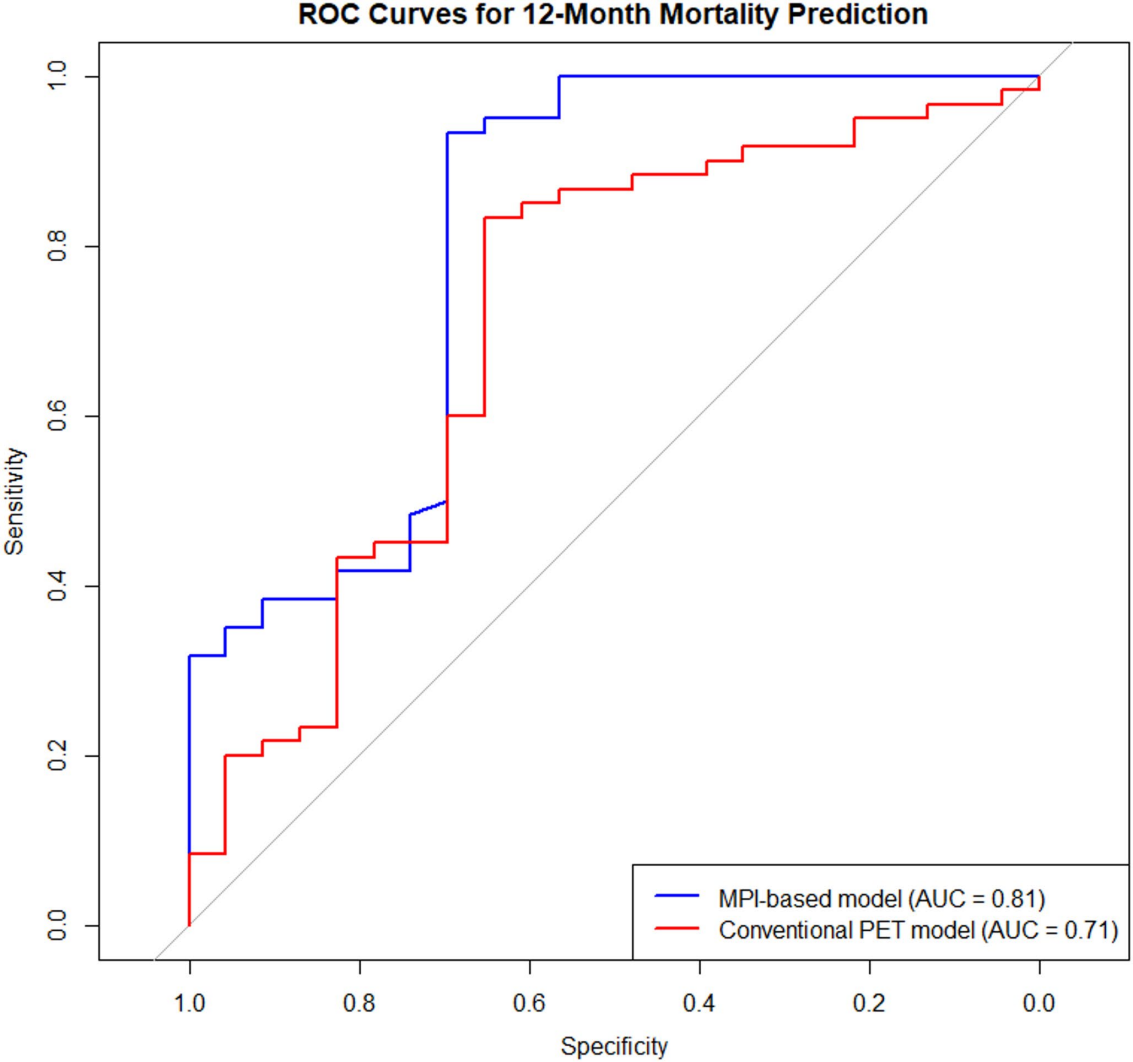
Receiver operating characteristic (ROC) curve analysis for prediction of 12-month all-cause mortality. The Pseudotime-based model demonstrated superior discriminative performance compared with the conventional PET-derived model, with a significantly higher area under the curve (AUC). Differences between ROC curves were assessed using DeLong’s test

The discriminative performance of the MPI-based model was superior to that of the conventional PET-based model incorporating standard imaging parameters (AUC 0.81 vs. 0.71) (Table 4) (Fig. 3). Although individual conventional PET parameters lost statistical significance in multivariable analysis due to collinearity, their combined model retained moderate discriminative ability. The difference in AUCs was statistically significant (ΔAUC = 0.10, 95% CI: 0.03–0.17; *p* = 0.002 by DeLong’s test) and remained consistent after bootstrap-based internal validation.

**Table 4:** The results of multivariate logistic regression

Model	Variable	OR (95% CI)	*p*-value	Model AUC
**Conventional Model**	tMTV	1.000 (1.000–1.001.000.001)	0.346	0.71
	DmaxVox	1.010 (0.980–1.041)	0.511	
	Bone Metastasis	1.663 (0.383–7.217)	0.497	
**MPI-Based Model**	MPI (per 0.1 unit)	1.87 (1.41–2.62)	< 0.001	0.81
	Bone Metastasis	5.988 (0.907–39.549)	0.063	

MPI: Metabolic Progression Index; tMTV: Total Metabolic Tumor Volume; DmaxVox: Maximum Euclidean distance between the two furthest voxels; AUC: Area Under the Curve; CI: Confidence Interval; OR: Odds Ratio

### Incremental prognostic value of MPI

Likelihood ratio testing demonstrated that replacing conventional PET-derived parameters with MPI resulted in a significant improvement in model fit (ΔDeviance = 22.8, *p* < 0.001), indicating substantial incremental prognostic value beyond traditional tumor burden metrics, despite partial overlap with spatial dissemination measures.

### Internal validation and risk profile analysis

Internal validation using bootstrap resampling (1000 iterations) demonstrated stable model performance, with an optimism-corrected AUC 95% CI ranging from 0.70 to 0.92.

**Fig. 4:**
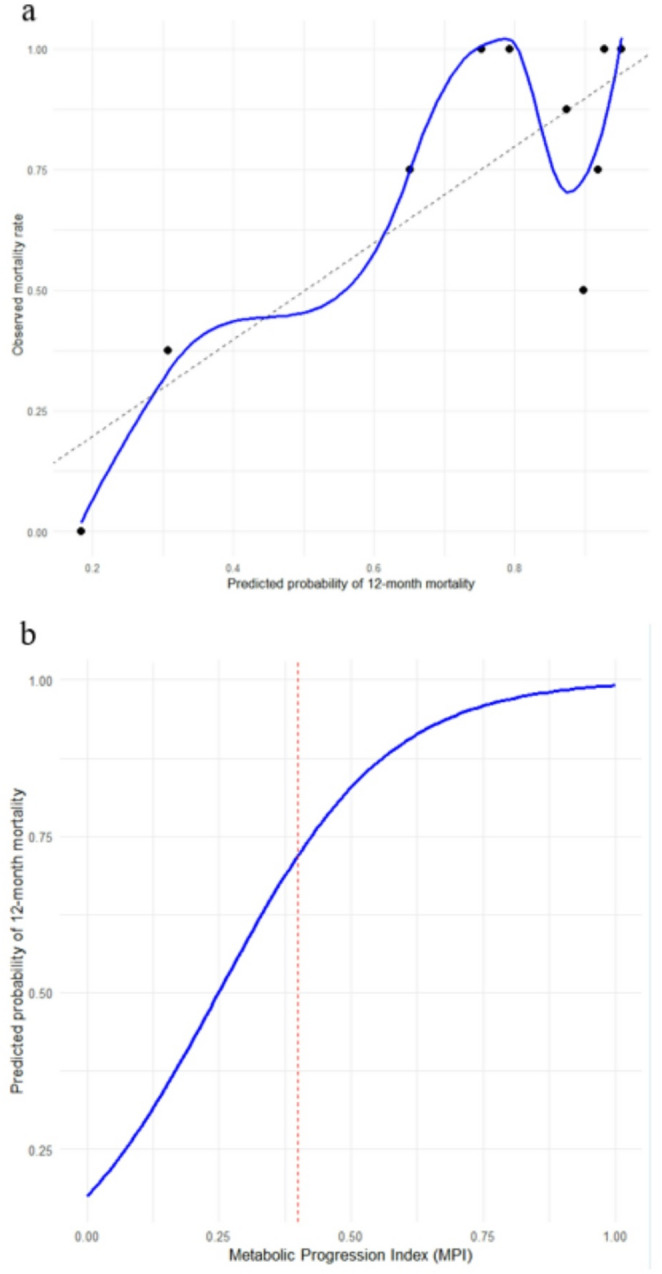
(a) Calibration plot illustrating the agreement between predicted and observed mortality probabilities. The dashed line represents perfect calibration, while the solid line shows the locally weighted scatterplot smoothing (LOWESS) (**b**) Risk profile curve showing the relationship between MPI and the model-predicted probability of 12-month mortality. Predicted risk increases nonlinearly across the MPI spectrum, with a region of steeper slope at higher MPI values; this pattern reflects the shape of the fitted risk function and is not intended to indicate a clinical threshold or cutoff

Risk profile analysis revealed a non-linear relationship between MPI and the predicted probability of 12-month mortality (Fig. 4). While mortality risk increased progressively across the MPI spectrum, a steeper rise in predicted risk was observed beyond MPI values of approximately 0.4. This observation is presented to illustrate the shape of the model-derived risk function rather than to propose a specific clinical cutoff.

### Time-to-Event survival analysis (Cox regression)

In univariable Cox regression, MPI was strongly associated with overall survival (HR = 3.31, 95% CI: 1.71–6.42, *p* < 0.001; C-index = 0.68), outperforming conventional PET-derived metrics such as tMTV (HR = 1.34, *p* = 0.007; C-index = 0.65) and DmaxVox (HR = 1.01 per mm, *p* = 0.01; C-index = 0.62). In multivariable Cox models including both tMTV and DmaxVox, neither parameter retained independent prognostic significance, consistent with strong multicollinearity between volumetric and spatial dissemination metrics. When bone metastasis status was added to the conventional PET-based model, none of the covariates remained independently significant, and overall model discrimination remained modest (C-index = 0.66). When modeled on its original 0–1 scale, MPI was associated with a HR of 3.31 (95% CI: 1.71–6.42, *p* < 0.001). For clinical interpretability, we additionally report effects per 0.1-unit increase (HR = 1.09 per 0.1 unit, 95% CI: 1.02–1.15).

### Internal validation and stability of MPI ordering

Subsampling-based robustness analysis demonstrated highly stable patient ordering along the MPI axis. Across 300 iterations using 80% random subsamples, the median absolute Spearman correlation between subsampled and full-cohort MPI rankings was 0.97, indicating high robustness of patient ordering to sampling variability.

To further evaluate whether the derived MPI is overly driven by spatial dissemination features more broadly, MPI was additionally re-derived after excluding both DmaxVox and Dmax from the feature set. The resulting dissemination-free MPI remained strongly concordant with the original index (Spearman’s ρ = 0.95; Pearson’s *r* = 0.89), indicating preservation of patient ordering along the latent severity axis. These findings further support that MPI is not a trivial reparameterization of spatial dissemination alone, but reflects an integrated phenotype arising from volumetric burden, intensity-based descriptors, and inter-lesional heterogeneity.

Alternative plausible root definitions (e.g., selecting the patient with minimal tMTV alone) resulted in highly concordant patient ordering (Spearman’s ρ > 0.96), indicating low sensitivity of the inferred trajectory to root selection.

### Additional comparison with simplified nonlinear PET models

To evaluate whether nonlinear combinations of tumor burden and dissemination metrics could approximate the prognostic information captured by MPI, we additionally constructed a simplified nonlinear logistic regression model incorporating second-order polynomial terms of tMTV and DmaxVox. This model demonstrated only modest discrimination (AUC = 0.65), substantially inferior to both the conventional PET-based multivariable model (AUC = 0.71) and the MPI-based model (AUC = 0.81). These findings indicate that neither linear nor nonlinear combinations of tumor burden and dissemination alone adequately recapitulate the multidimensional disease architecture captured by the diffusion-based latent trajectory.

## Discussion

In our study, we developed and validated a diffusion map–based, pseudotime-derived imaging biomarker—MPI—from baseline 18 F-FDG PET/CT in patients with extensive-stage SCLC. MPI demonstrated strong prognostic value for both 12-month all-cause mortality and overall survival, outperforming conventional PET-derived volumetric, metabolic, and spatial parameters across both classification- and time-to-event–based analyses. These findings support the concept that advanced SCLC is better represented as a continuous radiological severity spectrum rather than by isolated imaging descriptors.

MPI is intended not to replace established PET metrics such as MTV, TLG, or dissemination measures, but to integrate them into a unified representation of metastatic disease architecture. Although conventional PET parameters are biologically meaningful, they largely provide unidimensional summaries of a heterogeneous and systemic process [25]. In our cohort, strong collinearity among these parameters limited their combined use in multivariable models and motivated an integrative strategy. Notably, a simplified nonlinear model based solely on tumor burden and dissemination showed inferior performance compared with the MPI-based model, indicating that neither linear nor nonlinear combinations of conventional metrics adequately capture the multidimensional disease architecture encoded by the diffusion-based latent trajectory. Consistent with this interpretation, MPI retained prognostic relevance in time-to-event analyses, whereas conventional PET parameters lost significance when modeled jointly.

Clinically, MPI may serve as a continuous imaging-based measure of metastatic disease severity rather than a binary threshold. At baseline staging, MPI could support risk-adapted patient stratification by identifying individuals with highly complex and widely disseminated metabolic disease who may benefit from intensified follow-up, earlier response assessment, or prioritization for enrollment in clinical trials evaluating novel or intensified treatment strategies. In research settings, MPI provides a harmonized covariate that captures whole-body disease complexity, reducing reliance on multiple highly collinear PET parameters and facilitating analyses of treatment-effect heterogeneity. In longitudinal studies, changes in MPI across serial PET/CT scans could be explored as a marker of global disease trajectory, although this application requires prospective validation.

MPI represents a latent phenotypic continuum inferred from cross-sectional baseline imaging, whereby patients are ordered according to similarity in metabolic burden, spatial dissemination, and inter-lesional heterogeneity. The term “pseudotime” is used to denote a geometric ordering on a nonlinear manifold rather than true biological progression. Whether movement along this axis reflects temporal disease dynamics remains to be established through longitudinal imaging or biological validation.

The strong association between MPI and spatial dissemination metrics highlights the importance of anatomic spread in metastatic severity. However, several observations argue against MPI being merely a rescaled dissemination measure. Feature-wise analyses demonstrated that the dominant diffusion component was associated not only with dissemination but also with inter-lesional heterogeneity and global tumor burden parameters, supporting a multidimensional interpretation of the latent axis. Moreover, exclusion of dissemination features during MPI construction preserved the overall ordering of patients, indicating that the trajectory is not driven by a single dominant feature family. Together, these findings support MPI as a higher-order descriptor that contextualizes spatial spread within whole-body metabolic burden and heterogeneity.

The robustness of the inferred latent ordering was supported by subsampling-based stability analyses, indicating that the MPI axis is not a cohort-specific artifact driven by sampling variability. This is particularly relevant given the modest cohort size and addresses a key methodological concern in unsupervised trajectory inference.

Bone metastasis did not retain independent prognostic significance after adjustment for MPI, although its effect direction remained consistent with worse outcomes. This attenuation likely reflects shared prognostic information between skeletal involvement and the global metabolic–spatial disease complexity captured by MPI, suggesting partial overlap rather than loss of clinical relevance.

Risk profiling indicated a nonlinear relationship between MPI and predicted mortality, reflecting the continuous nature of risk along the latent severity spectrum rather than a clinically actionable threshold. To facilitate interpretability without implying cutoffs, effect estimates were expressed per incremental change in MPI.

This study has limitations, including its retrospective single-center design and lack of external validation. Biological correlates of MPI were not assessed, and clinical or laboratory biomarkers were not incorporated, as the primary objective was to derive an outcome-agnostic imaging-based severity axis. Fixed SUV threshold segmentation may underestimate low-uptake or very small lesions; however, the use of a uniform threshold across patients supports internal consistency for relative ordering along the latent trajectory. Future studies should validate MPI in independent cohorts, evaluate harmonization across scanners and segmentation strategies, and explore integration with molecular, immunological, and clinical variables.

In conclusion, integrating high-dimensional 18 F-FDG PET/CT features through a diffusion map–based pseudotime framework yields prognostic information beyond conventional PET-derived metrics in extensive-stage SCLC. MPI captures a latent dimension of metastatic disease architecture within a single continuous coordinate and represents a potentially robust, automatable imaging biomarker for risk stratification in aggressive metastatic cancers.

## Data Availability

The datasets generated during and/or analyzed during the current study are not publicly available due to patient privacy concerns but are available from the corresponding author on reasonable request.
